# Electron Spin Relaxation in Carbon Materials

**DOI:** 10.3390/ma15144964

**Published:** 2022-07-16

**Authors:** Damian Tomaszewski, Krzysztof Tadyszak

**Affiliations:** Institute of Molecular Physics Polish Academy of Sciences, ul. Mariana Smoluchowskiego 17, 60-179 Poznań, Poland; damian.tomaszewski@ifmpan.poznan.pl

**Keywords:** EPR, spin–spin relaxation time, spin–lattice relaxation time, phase memory time, graphene, graphene oxide, reduced graphene oxide, diamond, anthraxolite, anthracite, activated carbon fibers

## Abstract

This article focuses on EPR relaxation measurements in various carbon samples, e.g., natural carbons—anthracite, coal, higher anthraxolites, graphite; synthetically obtained carbons—glassy carbons, fullerenes, graphene, graphene oxide, reduced graphene oxide, graphite monocrystals, HOPG, nanoribbons, diamonds. The short introduction presents the basics of resonant electron spin relaxation techniques, briefly describing the obtained parameters. This review presents gathered results showing the processes leading to electron spin relaxation and typical ranges of electron spin relaxation rates for many different carbon types.

## 1. Introduction

When no action is undertaken, the spin system is in its ground state. This means that most molecules have the lowest energy. Only a small fraction is excited to energetically higher laying states, with probabilities described by a Boltzmann distribution. The most general description of spin relaxation is the process of returning the excited spins to their natural state described by the Boltzmann distribution. Depending on the energy dissipation pathway, some fundamental couplings between applied photons and nuclei, electrons, and phonons can be studied [1,2]. All this gives characteristic relaxation times, mechanisms, and energy dissipation efficiency. The relaxation process allows for a better understanding of interdependencies between the participants in this process and its quantum mechanics. Carbon-based materials exist in many forms, e.g., graphene [3,4,5], graphene oxide quantum dots [6], nanotubes [7], graphene oxide (GO) [7,8,9,10], GO fibers [11], GO foams [12], diamonds [13,14,15,16,17,18,19], and are required in the fields of energy applications, quantum computing/spintronics and biology [20,21]. The source of an electron paramagnetic resonance (EPR) signal can be related to conduction electrons, e.g., anthracite [22], or localized paramagnetic states, e.g., GO [8] and graphene [23,24] across different carbon materials. Even across the same sample, the EPR signal can come from conduction electrons for large flakes and localized centers for smaller than 1 μm^2^ flakes, Ćirić et al. [25,26]. A nontrivial temperature dependence of the EPR signal intensity indicates antiferromagnetic correlations between localized paramagnetic centers, Diamantopoulou et al. [27]. Kempinski et al. [28] suggested that the GO EPR signal comprises two Lorentzian lines where one belongs to localized charge carriers. Augustyniak et al. explained a substantial increase in normalized EPR signal intensity at low temperature due to Anderson localization of charge carriers [29]. Other effects such as Rabi splitting [30], antiferromagnetic interactions of defects on graphene’s surface, and ferromagnetic correlations on the zigzag edge [31,32,33] were also reported. 

## 2. Background

It is worth mentioning that there are two main methods of exiting the system: resonant and nonresonant. An example of the latter is the measurement of dynamic magnetic susceptibility (A.C. susceptibility) [34]. However, this technique does not address a two-level directly. Due to this feature, the result is an average. Furthermore, to obtain a satisfactory signal-to-noise ratio, the nonresonant technique uses concentrated paramagnetic systems instead of diluted ones. In this article, we will be dealing only with resonant relaxation processes. The system, which usually is a diluted paramagnet, is excited from its ground state by an impulse of electromagnetic radiation whose frequency fulfills the resonance condition caused by the external magnetic field. This method addresses specific transitions between the spin system, and the time it takes for the spins to return to the initial Boltzmann distribution is measured. This article focuses only on the relaxation in electron paramagnetic resonance spectroscopy, where usually microwave pulses are absorbed by the electron spins. Energy is dispersed in the form of phonons (in crystals), allowing the system to relax. This excess energy is released at a specific time, which defines the sample. In the field of electron magnetic resonances, three essential times describe the system. Similar, but not always identical, times exist in nuclear magnetic resonance. Although, due to weaker coupling of nuclei with electrons and further phonons, relaxation mechanisms and time scales are different than in EPR (milliseconds instead of nanoseconds). 

First is the spin–lattice relaxation time T_1,_ which describes the return time of the initial magnetization pushed out of the equilibrium position, which is the direction of the external magnetic field. The first method of estimating this time is the microwave saturation technique (Bloch method) used with a continuous-wave spectrometer (CW-EPR). After developing pulse EPR spectrometers, relaxation time could be measured by saturation or inversion magnetization recovery on an echo or free induction decay (FID) [35]. 

Next is the spin–spin relaxation time T_2,_ the characteristic time of free induction (magnetization) decay that appears after one π/2 pulse. It is also the time that magnetization spends in the *xy* plane (after excitation from the initial *z*-direction) until it spreads evenly in the plane and stops being detectable. If the line is homogeneously broadened, the T_2_ time can be estimated by a Bloch relation from line width in CW-EPR experiments [36], in another case from induction decay appearing after one microwave pulse or from echo decay.

The last time is the phase memory time T_m_. It tells how long the spin states remain coherent. It describes the decay of the echo amplitude after increasing the dwell time between two pulses. If the relaxation rate T_2_^−1^ is proportional to the CW-EPR line width, then the T_m_^−1^ rate is proportional to the line width of a single spin packet hidden under the unresolved CW-EPR line. 

All times mentioned by the cited authors’ (T_1_, T_2_*, T_m_) are presented as relaxation rates (T_x_^−1^). The indexes indicate the method of measurement and not a new entity. The description is as follows:

- T_1CW_^−1^, T_1echo_^−1^, T_1FID_^−1^ are spin–lattice relaxation rates obtained from continuous saturation wave (CW-EPR), echo or FID inversion, or saturation recovery in pulse experiments, respectively. T_2CW_^−1^*, T_2FID_^−1^*, and T_2echo_^−1^* are spin–spin relaxation times obtained from CW-EPR lines, FID, and echo, respectively.
- T_m_^−1^ phase memory relaxation rate obtained in pulse EPR experiment by measuring the echo intensity decrease with the increase in dwell time between pulses.
- T_D_^−1^ diffusion and relaxation rate based on the Dyson theory [36,37,38]. Diffusion time is the time necessary for an electron to pass the length of the skin depth.
- T_1 Dys_^−1^ = T_2 Dys_^−1^ Dyson theory-based relaxation time is the time between two scattering events in the conductive samples in which the EPR signal originates from conduction electrons. In Dyson’s theory, spin–lattice T_1 Dys_^−1^ and spin–spin relaxation T_2 Dys_^−1^ rates are equal.

Many allotropes of carbons exist, i.e., graphite, graphene, fullerene, and diamond, whose properties such as conductivity and magnetism differ strongly, although all being purely carbon structures. This review is focused on electron spin relaxation phenomena observed using EPR spectroscopy. The primary distinction between these carbon forms is in the structure, leading to differences in the source of the EPR signal (radicals, defects, edge states, and conduction electrons), level of conductivity, and preparation before measurement. Depending on the preparation, especially in the case of conductive samples, different results can be obtained.

One of the first signal sources in conductive materials and carbon (e.g., graphene) was conduction electrons. The first conduction electron signal was reported by Griswold in 1952 [39]. This marked the beginning of a new field of study, which nowadays is known as Conduction Electron Spin Resonance (CESR). The influence of spin–orbit coupling on the resonance line was described by Elliot in 1954 [40]. In 1955, Dyson [37] and Feher [38] published cooperatively theoretical and experimental articles, respectively, describing the signal form and comparing the theoretically obtained curves with experimental results. Dyson used Green’s functions method to describe the line shape. Simpler approaches were proposed in the following years [41,42,43,44]. The observation of the CESR phenomenon in materials with high electrical conductivity is technically difficult, and the description of the spectra requires consideration of a number of additional effects. The most important of these is the effect of damping of the electromagnetic field oscillations, which restricts the resonance phenomenon to the skin depth δ, the characteristic length at which the eddy currents reach e^−1^ of its surface value. The source of the signal lies in the conduction electrons located close to the surface in a magnetic field gradient induced by the skin effect. It is possible to observe symmetrical or asymmetrical resonance lines dependent on conditions, e.g., electrical conductivity and the sample size. In the case of conductive samples, Dyson’s theory predicts that T_1_ and T_2_^*^ relaxation times are equal. This theory found its use in explaining graphite [45] and graphene spectrum [23]. Linear temperature dependence of spin–lattice relaxation was explained by Korringa [46] or Barnes relaxation [47] occurring through conduction electron. Electron spin relaxation in amorphous solids (e.g., polymers, conductive polymers, glasses) and disordered solids (e.g., graphene oxide, reduced graphene oxide, or other graphene-based structures) have not been studied well, basically due to a lack of data about phonon propagation and the spin relaxation mechanism in amorphous structures that are not mentioned in cases of electrically conductive amorphous structures, where relaxation can also go over conduction electrons together such as through localized phonons. The latter are phonons that are limited to only a small part of the sample, characterized by a mean free path (l) smaller than the dimensions of the sample (L) (l << L) [34]. Relaxation in amorphous solids, e.g., disordered carbons, is connected with phenomena such as tunneling of two-level systems, soft local oscillators, and localized phonons [48]. The typical relaxation mechanism in such structures is based on a tunneling two-level system (TLS) [48,49]. TLS is a theoretical concept that assumes that the potential energy surface of an amorphous solid contains many shallow asymmetric local minima, which can be approximated by a collection of two-level double wells. In amorphous materials, such potential minima can be imagined as two local conformations of molecules or two possible sites for some atoms [48,50].

## 3. Results 

### 3.1. Natural Carbons

**Higher anthraxolites,** e.g., Shungite-I (type 1), are well-conductive natural carbons (C ~ 98%, σ ~ 1.2–2.7 kSm^−1^ [51]), classified as the highly metamorphosed bitumen. Bulk samples show an asymmetrical Dyson line, which changes to a symmetric Lorentz line shape if the sample is fine powdered. From the fit with Dyson formula, direct spin–lattice as well spin–spin relaxation rates are gained T_1_^−1^ = T_2_^−1^ = 35.2 MHz along with diffusion rate T_D_^−1^ = 57.8 MHz [36] (at 5.2 K, for CESR see [37,51]). Results can differ depending on deposit and coal type.

**Anthracite** is a most metamorphosed graphitizable coal, with a high carbon content of 91–98 wt% and shallow hydrogen content lower than 3 wt%. Anthracite shows room-temperature-specific conductivity of about 100 Sm^−1^ (skin depth δ of 0.5 mm). The slight decrease in anthracite particle size can cause symmetrization of the EPR signal. The line symmetrizes due to a decrease in conductivity with the decrease in temperature related to hopping-type conductivity. All above makes it hard to correctly estimate relaxation times using the Dyson theory [51]. 

In the case of powder samples, the signal can be resolved into two Lorentz lines. It is assumed that at least one of them corresponds to the interacting conducting electrons, which form a conduction band and show Pauli paramagnetism. Curie-type contribution to susceptibility can be contributed by conduction electrons isolated in some small regions [22]. The two-component structure of the powder EPR spectra was explained by the size distribution of grains and a more effective scattering of conduction electrons by the surface roughness in smaller grains. Additionally, Rabi splitting, which appears due to the strong coupling between electron spins and the cavity effects, was observed. This effect can be used to estimate the spin number of paramagnetic centers in the sample [30]. 

**Argonne Premium Coal** is considered a medium rank bituminous coal with a carbon content of 82% to 63%. It is a common practice to specially prepare the sample before measurement. Usually, it is vacuum annealing >140 °C with previous chemical treatment. The obtained fractions (hole coal = extract + residue) can be treated separately, giving different results from the untreated coal. Consequently, the surviving extract radicals exhibit more extensive heteroatom content than the whole original coals. 

Usually, samples show a two-component EPR spectrum (Lorentz lines). Saturation recovery was applied to detect the broad signal and FID for the narrow line. The presence of two types of hydrogen groups was detected. The T_1echo_^−1^ relaxation rate was in the range of 6.25 kHz–15.6 kHz in hole coal. A faster relaxation rate T_1_^−1^ is observed when samples have more carbon content [52]. In the inertinite fractions of Argonne Premium Coal, the spin–spin relaxation rate was in the range T_2FID_^−1^ ~0.37–2.8 MHz [53], and spin–lattice relaxation rate T_1echo_^−1^ was in the range 2–67 kHz (saturation recovery curves). Two-component spectrum with two distinct phase memory times T_m_^−1^ in the range 0.83–2.8 MHz was observed [54]. 

**Vitrinite Coal Maceral** was studied by pulse techniques reaching values of T_1_^−1^ in the range 14–268 kHz (inversion recovery) and T_m_^−1^ of 0.6–4 MHz from the lowest to the highest rank of macerals, respectively [55]. 

### 3.2. Glassy Carbons

Another group of carbon materials consists of **pyrolyzed organic compounds,** which form a group of **synthetic carbons** resembling the carbon structure but at the same time have higher carbon content with fewer contaminations than in natural coal. Examples of these structures are as follows:

**Sugar char** was studied using the continuous-wave saturation method [56]. The authors discuss the differences in the saturation behavior of chars in a vacuum and in the air with a focus on oxygen, which can interact with carbon both chemically and physically, changing the spin–lattice relaxation time (no relaxation times were evaluated). This sensitivity to oxygen is visible in line shape broadening and was used for contrasting images in ex vivo EPR imaging [57]. 

Pyrolysed **butene-1** and **n-butane** on the alumina surface [58]. Continuous-wave saturation method allowed obtaining the relaxation rates as follows: T_2CW_^−1^ = 2.16 MHz and T_1CW_ ^−1^ in the range 9.09 kHz–400 kHz. It was found that the relaxation rate increases at room temperature with an increase in carbon content (from 3.6% to 22.2%).

After carbonization of polyparaphenylene at temperatures 650 °C and 700 °C, authors observed a single broad peak for the first sample (ΔH_pp_ = 5 G) with non-Lorentzian line shape, and two peaks for second sample (ΔH_pp_ = 3.9 G, 0.47 G) described by Lorentzian derivatives. In higher temperatures, only the narrow signal remained. Multiple samples differentiated by carbonization temperature show spin–lattice relaxation rates T_1CW_^−1^ in the range 4.4–15.1 MHz and T_2CW_^−1^ in the range 12.4–5.2 MHz (2900 °C–650 °C, respectively). Spin lattice relaxation rate increases with the carbonization temperature but the spin–spin relaxation rate changes in the opposite direction [59].

### 3.3. Activated Carbon Fiber

**Activated carbon fibers (ACF)** are the outcome of the pyrolysis of polyacrylonitrile (PAN) and other polymers. After activation, the specific surface area can reach 3000 m^2^/g [60]. The EPR signal is strongly dependent on the gas adsorbed on the surface. Surface defects, ad-atoms, and dangling bonds can be the source of the EPR signal. Usually, two signals are observed with two contributions to spin–lattice relaxation rate, where one is mediated by conduction electrons [61,62], where T_1CW_ is well-coincident with the change in the electric conductivity and the chemical insulation of ACFs and understood by the Korringa relation scheme. The second contribution depends on the adsorbed gas or molecules, although the effect of contaminated oxygen should be carefully eliminated. The microwave power saturation curves depend on the presence of gas adsorbed. It is harder to saturate the EPR line when higher gas pressure is around the sample (e.g., He). The experiment suggests a microwave energy transfer from the sample to gas and prolonged relaxation. Obtained relaxation rates are as follows: T_1CW_^−1^ = 0.1 MHz at 0 Torr pressure and rises to 1.1 MHz at 10 Torr [60]. Other ACF samples with pore size 1.2–1.4 nm with and without nitrobenzene absorbed showed electron spin echo in a very narrow temperature range. Before the adsorption of the nitrobenzene guest, the ACF samples were evacuated at 200 °C at 10^−4^ mbar for 1 h to ensure that the pores were empty. The samples for ESR measurements were degassed and sealed under a vacuum to avoid contact with atmospheric oxygen. Nitrobenzene samples showed slightly higher relaxation T_1_^−1^ rate ACF T_1_^−1^ 0.002–0.2 (12–35 K) and lower T_M_^−1^ rate 0.37–1.25 (12–35 K) than empty ACFs [49].

### 3.4. Graphite

Three-dimensional crystalline, hexagonal structure of carbon **graphite** is imagined first when mentioning carbon. The structure is electrically conductive, and the EPR signal is asymmetrical and can be described in the framework of Dyson’s theory. As an example, neutron-irradiated graphite can be presented, whose relaxation rates are equal to T_1CW_^−1^ = T_2CW_^−1*^~ 50 MHz at 300 K. T_1_ is calculated from the perpendicular component of the linewidth and dominated by carrier scattering via spin–orbit interaction. The decrease in line asymmetry induced by irradiation [63] is connected with increasing structural disorder and decreasing electrical conductivity.

**Graphene** is a two-dimensional carbon structure that has gained huge interest in recent years [64]. This is partly due to its large charge carrier’s mobility and hopes of application in high-frequency transistors, but also because the magnetic interactions in graphene are not trivial. It was reported that the paramagnetic surface defects could bind antiferromagnetically while being coupled by the RKKY (Ruderman–Kittel–Kasuya–Yosida) interaction through conduction electrons [32]. The zigzag edge states can be coupled ferromagnetically [33], which was studied by FMR [31]. The reported relaxation rates T_1FID_^−1^ (1-5 MHz) and T_2FID_^−1^ (2–5 MHz) are close to each other in the range of 5–140 K, respectively [23]. The temperature spin–lattice relaxation dependence in this range was described by the Korringa and Bloch–Hasegawa linear relationship. Using the Bloch equation for the estimation of T_2CW_^−1*^ relaxation rate, similar results were obtained for the pulse method: 1.75–4.5 MHz in the temperature range 5–300 K, respectively. Spin dynamics in graphene-based materials can vary, e.g., in graphene nanoribbons: two slow spin–lattice relaxation rates were observed T_1_^−1^ 0.045–0.173 MHz (5–290 K) and 0.0049–0.018 MHz (5–290 K) while T_M_^−1^ was 1.9-1.7 MHz (5–290 K) and 34.9–27.5 MHz (5–290 K) [65], graphene T_1_^−1^~ T_2_^−1^*~3 MHz [23]. In general, across literature, authors have observed one (single-flake graphene) [23,32,33] or two spin–lattice relaxation processes (ribbons) [65]. 

### 3.5. Graphene Oxide

Due to many oxygen-rich hydrophilic groups on its surface, **graphene oxide** is an essential material from an applicational point of view [64]. A single Lorentz line can resolve the EPR line. The spin–lattice relaxation rate T_1FID_^−1^ in graphene oxide increases from 19 kHz (52 ms) at 5 K to 6.54 kHz at 240 K (0.153 ms). Compared with reduced graphene oxide and other carbon materials, GO exhibits a highly long spin–lattice relaxation time. The phase memory rate is about 1 MHz at 240 K (1 μs), and it changes non-monotonically with lowering the temperature and reaches its maximum of 0.45 MHz at 5 K (2.2 μs) [66]. The temperature dependence can be described with equation T_1_^−1^ = *AT* + *BT*^5^, describing the direct and multiphonon Raman relaxation process [66]. 

### 3.6. Reduced Graphene Oxide

The reduction of graphene oxide enables the production of a chemically derived form of graphene known as **reduced graphene oxide (rGO)**. The properties of rGO strongly depend on the method used for the reduction. Partially reduced graphene oxide can show one component of the signal with relaxation rate T_1echo_^−1^ 17.5–22.5 MHz in the temperature range 100–260 K [12], respectively, or two components of T_1Aecho_^−1^ = 3.36 MHz, T_1Becho_^−1^ = 0.4 MHz at 5 K and goes to T_1Aecho_^−1^ = 26.65 MHz, T_1Becho_^−1^ = 1.16 MHz at 200 K, T_mA_^−1^ = 4.03 MHz, T_mB_^−1^ = 0.68 MHz (4.67 and 0.67 MHz at 200 K, respectively), T_2_^−1^ = 25.1 MHz at 5 K (30.3 MHz at 200 K). Data in the abovementioned cases were obtained with the inversion recovery technique. Hydrothermally reduced graphene oxide shows two components of T_1echo_^−1^ at temperatures T < 20 K, which can be described with formulas T_1A_^−1^ = 3.1 × 10^5^ + 10^8^ × T^2^ and T_1B_^−1^ = 3.3 × 10^4^ + 51 × T^2^ [67]. At 20 K, one can obtain values T_1Aecho_^−1^ = 0.35 MHz and T_1Becho_^−1^ = 0.054 MHz. Above 20 K, the second type of the paramagnetic center disappears. Temperature dependence of T_1_^−1^ ~ T^2^ is observed for disordered or amorphous materials and indicates the phonon bottleneck [67]. At temperatures below 20 K, values of the T_m_^−1^ depend slightly on temperature. An increase in temperature from 5 to 20 K causes changes in T_mA_^−1^ from 4.03 MHz to 3.76 MHz, and T_mB_^−1^ from 1.06 MHz to 0.97 MHz. Above 20 K, T_mA_^−1^ decreases rapidly to 0.24 MHz at a temperature of 100 K.

Other authors for commercially obtained reduced graphene oxide observed two components’ spectra characterized by the two spin–spin relaxation time T_2CW_^−1^. At the temperature of 290 K, the values T_2A_^−1^ = 77 MHz and T_2B_^−1^ = 250 MHz were obtained; at the temperature of 80 K, T_2A_^−1^ = 50 MHz and T_2B_^−1^ = 250 MHz, respectively [68]. In the case of rGO obtained with reduction using hydrazine hydrate, the following relaxation times were observed at 300 K: T_1CW_^−1^ = 180 MHz and T_2CW_^−1^ = 186 GHz [69]. The spin–spin and spin–lattice relaxation times are enhanced by strong reduction, which causes the increase in conductivity and crystalline order where phonons can easier propagate compared with GO.

### 3.7. Diamond

Many different paramagnetic centers occur naturally after irradiation in **diamonds.** A review summarizing all of them can be found here [70]. The early work of Bell and Leivo [71] shows the estimated spin–lattice relaxation rate in semiconducting diamonds of the order 100 MHz (at 300 K) using the Bloch method [72]. The EPR center was associated with p-type donors. In thin diamond films, the spin–lattice relaxation rate is T_1CW_^−1^ ~1 MHz (300 K, line saturation). It went up to 10 MHz at around 500 K and was found to depend on the morphology of the films. Samples show independent spin–lattice relaxation rate for T < 100 K and T^α^, where α = 3 for ball shape diamonds and α = 5 for the sample with facet morphology [73]. The study of Fanciulli et al. [74] performed on thin diamond film doped with nitrogen showed a paramagnetic defect giving a single EPR line having a Lorentz shape (~6.5 G) and coupled by hyperfine interaction to the nitrogen atom. Using the saturation technique, T_1_^−1^ rate was obtained having a room temperature value 100 kHz, which decreased to 1 kHz at 10 K [74].

### 3.8. Fullerene

A **fullerene** molecule consists of carbon atoms linked by single and double bonds to form a closed or partially closed lattice, with fused rings of five to seven atoms. The fullerene anions C_60_^1−^ and C_60_^3−^ can be generated electrochemically in 4:1 toluene:acetonitrile or DMSO containing 0.1 M tetrabutylammonium hexafluorophosphate [75,76]. Anion fullerene C_60_^1−^ has one unpaired electron, which makes it paramagnetic (C_60_^2−^ is diamagnetic) and C_60_^3−^, although the signals are hard to distinguish. In cases of C_60_^1−^ and C_60_^3−^, relaxation times T_1_ were dependent on the magnetic field position set (low or high part of the magnetic field of the resonance line) explained by the Jahn–Teller distortion [75]. For the C_60_^1−^ anion, spin–lattice relaxation rate T_echo1_^−1^ = 3.5 kHz (at 8 K, saturation recovery) low field end up to 18 kHz high field spectrum and the phase memory time T_m_^−1^ = 0.45 MHz (at 15 K, single exponential function) up to 3.5 MHz at the high field end of the spectrum in frozen toluene–acetonitrile solution [76]. 

Saturation recovery measurements on C_60_^1−^ in 4:1 toluene:acetonitrile containing 0.1 M TBAPF6 allowed to determine the relaxation rates by fitting the experimental data to a single exponential: T_echo1_^−1^ = 1.25–18 kHz (at 8 K), T_echo1_^−1^ = 2.5–80 MHz (17 K), while T_m_^−1^ = 2–12.5 MHz (45 K), T_m_^−1^ = 0.2–11 MHz (9 K). Saturation recovery curves T_echo1_^−1^ for C_60_^3-^ were not single-exponential decays, while T_m_^−1^ was single with T_m_^−1^ = 0.7–2.1 MHz (at 7 K) and T_m_^−1^ = 3–16 MHz (36 K) [76]. The samples showed wide distribution in relaxation rates at each point in the spectrum, and the question of two contributions in the relaxation time is still open [75]. Investigation of the dependence of the EPR signal intensity on the square root of microwave power for C_60_ shows the same order of magnitude of spin–lattice and spin–spin relaxation times, T_CW1_^−1^ = T_CW2_^−1^ ~ 100 MHz at room temperature [77]. The concentration of paramagnetic centers was estimated at 10^17^ spin/g, which corresponds approximately to 1 paramagnetic defect for 5000 fullerene molecules.

### 3.9. Carbon Nanotubes

**Carbon nanotubes (CNTs)** were produced by chemical vapor deposition using a Fe catalyst [78]. Defects were induced in MWNTs by acid digestion in a nitric and sulfuric acid mixture. Relaxation times were measured with the continuous-wave saturation method: T_1_^−1^ 6.7 kHz (<10 K) and rose to around 140 kHz (>125 K), while T_2_^−1^ was 1.5 MHz.

## 4. Conclusions

Carbon samples differ in structure, crystalline ordering, number of defects, their interactions, and purity. All mentioned carbon samples exhibited from one to three line components in EPR spectroscopy. These components can be related to surface defects missing carbon atoms, additional ad-atoms or functional groups, edge magnetic moments related to edge termination, or conduction electrons. Analysis of data in Table 1 shows that the slowest relaxation rate T_1_^−1^ is for natural coals, graphene oxide, and fullerenes. Relaxation rates are highest in two cases for well-developed crystalline structures diamond and graphene with additional high electrical conductivity. Higher spin–lattice relaxation rates are observed in samples with higher carbon content (e.g., natural carbons and glassy carbon) and higher carbonization temperature (glassy carbons). Relaxation rates, as well as the signal shapes, can be easily modified by surface chemistry or adsorption in the case of porous, large-surface-area samples (e.g., activated carbon). All of this suggests that relaxation mechanisms are related to phonons, which are the most critical relaxation mechanism in diamagnetic crystals, but also that the role of conduction electrons cannot be omitted. The increase in the spin–lattice relaxation rate between graphene oxide and reduced graphene oxide can suggest the additional influence of conduction electrons on spin–lattice relaxation.

## Figures and Tables

**Table 1:** Electron spin–lattice, spin–spin, diffusion, and phase memory relaxation rates for different carbon types.

Sample	T_1_^−1^ (MHz) (Temp.)	T_2_^−1^ (MHz) (Temp.)	T_m_^−1^ (MHz) (Temp.)	Ref.
**Higher anthraxolites**	35.2 (5.2 K)		T_D_^−1^ = 57.8 (5.2 K)	[36]
**Anthracite**	not measured			
**Argonne Premium Coal (APC)**	0.00625–0.0156	0.37–2.8	**---**	[52]
**Inertinite fractions of APC**	0.002–0.067	**---**	0.83-2.8	[53]
**Vitrinite Coal Maceral**	0.014–0.268	**---**	0.6-4	[55]
**Glassy carbon**	0.0091–0.4	2.16	**---**	[58]
**Glassy carbon**	4.4–15.1	12.4–5.2	**---**	[59]
**Activated carbon fibers**	0.1–1.1 pressure-dependent	**---**	**---**	[60]
**Graphite**	50		**---**	[63]
**Graphene**	1–5 (5–140 K)	2–5 (5–140 K)	**---**	[23]
**Graphene**	**---**	1.75–4.5 (5–300 K)	**---**	[23]
**Graphene**	3 (100 K)		**---**	[23]
**Graphene nanoribbons**	0.045–0.173 (5–290 K) 0.0049–0.018 (5–290 K)	**---**	1.9–1.7 (5–290 K) 34.9–27.5 (5–290 K)	[65]
**Diamond**	100 (300 K)	**---**	**---**	[72]
**Diamond films**	1–10 (300–500 K)	**---**	**---**	[73]
**Diamond doped with nitrogen**	0.01–0.100 (10–300 K)	**---**	**---**	[74]
**Fullerene**	0.0035–0.018 (15–240 K)	**---**	0.45–3.5 (15–240 K)	[75]
**Graphene oxide**	0.019–0.00654 (5–240 K)	**---**	0.45–1 (5–240 K)	[66]
**Reduced graphene oxide**	3.36–26.65 (5–200 K) 0.4–1.16 (5–200 K)	**---**	4.03–4.67 (5–200 K) 0.68–0.67 (5–200 K)	not published
**MWCNTs**	0.00666 (<10K) ~0.143 (>125 K)	1.5		[78]
**ACF**	0.002–0.2 (12–35 K)		0.37–1.25 (12–35 K)	[49]

## Data Availability

Not applicable.
